# Cutaneous Metastasis From Tonsillar Squamous Cell Carcinoma: Report and Review of the Literature

**DOI:** 10.7759/cureus.1122

**Published:** 2017-03-28

**Authors:** Omar Bari, Philip R Cohen

**Affiliations:** 1 School of Medicine, University of California, San Diego; 2 Department of Dermatology, University of California, San Diego

**Keywords:** carcinoma, cutaneous, metastasis, squamous, tonsil

## Abstract

Tonsillar squamous cell carcinoma, which represents 10% of head and neck malignancies, rarely manifests with cutaneous metastases; to date, only three prior patients with tonsillar squamous cell carcinoma have been reported to develop cutaneous metastases. We describe the clinical features of a 59-year-old man with squamous cell carcinoma of the tonsil who developed cutaneous metastases within his prior radiation port and review the literature of prior patients with cutaneous metastases from tonsillar squamous cell carcinoma. The PubMed database was searched for the following keywords: carcinoma, cutaneous, metastasis, squamous, and tonsil. The papers generated by the search and their references were reviewed. Cutaneous metastasis from tonsillar cancer is rare but should be considered in patients with a history of a squamous cell carcinoma of the tonsil; new skin lesions, both overlying the visceral malignancy and at more distant sites, should be biopsied since prognosis in these patients is poor. Management has thus far been palliative and should be individualized to the patient.

## Introduction

A new skin lesion in a patient with a history of cancer could be a cutaneous metastasis, particularly if it appears in an area of the body overlying the visceral malignancy [1]. The morphology of cutaneous metastases may vary, and lesions may also be found at distant sites. A retrospective review of 4,020 patients with metastatic cancer revealed that 10% of the patients developed cutaneous metastases; the most common malignancies manifesting with cutaneous metastases were breast cancer and melanoma [2]. Squamous cell carcinoma of the tonsil, which represents 10% of head and neck malignancies, may also manifest with cutaneous metastases [3]. However, only three prior cases of cutaneous metastases secondary to this malignancy have been reported to date [3-5]. Biopsy is warranted when there is suspicion for cutaneous metastasis, since spread of the visceral tumor to the skin is usually a poor prognostic marker [1, 4]. We describe the clinical features of a man with squamous cell carcinoma of the tonsil who developed cutaneous metastases in his prior radiation port and review the features of prior patients whose tonsillar squamous cell carcinoma metastasized to their skin.

## Case presentation

A 59-year-old Caucasian man with stage IV squamous cell carcinoma of the tonsil presented for evaluation ulcerated nodules on his neck and multiple erythematous papules and plaques on his proximal chest. The patient had no history of alcohol or tobacco use and was diagnosed with invasive squamous cell carcinoma of the right tonsil in September 2012. He was treated with three cycles of cisplatin along with radiation therapy to his neck and upper chest over two months with good response.

In 2014, recurrence of squamous cell carcinoma was discovered in his cervical lymph nodes. He was treated with bilateral selective neck dissection. In 2015, he developed severe dysphagia, and esophagogastroduodenoscopy with dilation was performed. Tracheostomy tube was placed due to airway compromise.

He underwent a second esophagogastroduodenoscopy with dilation three months later in July 2015 for continued dysphagia; during the procedure, an abnormal area on the posterior pharyngeal wall was noted. Subsequent direct laryngoscopy with biopsy revealed squamous cell carcinoma in-situ. However, the gross appearance of the lesion was suspicious for invasive disease; positron emission tomography–computed tomography (PET-CT) was consistent with local progression of his carcinoma.

In November 2015, he was treated with two cycles of carboplatin and paclitaxel chemotherapy. He was then transitioned to nivolumab; he received 16 cycles from December 2015 to July 2016. In August 2016, he was restarted on carboplatin and paclitaxel. After three cycles, the patient decided to discontinue chemotherapy in November 2016.

In January 2017, the patient presented to his otolaryngologist for routine tracheostomy tube change. He described a two-month history of a neck rash that was initially painful, had spread to his chest, and was vesicular in appearance. The otolaryngologist noted not only a malodorous discharge near the tracheostomy site, but also skin lesions that were suspicious for varicella zoster virus infection. The patient was referred to a dermatologist for evaluation.

**Figure 1:**
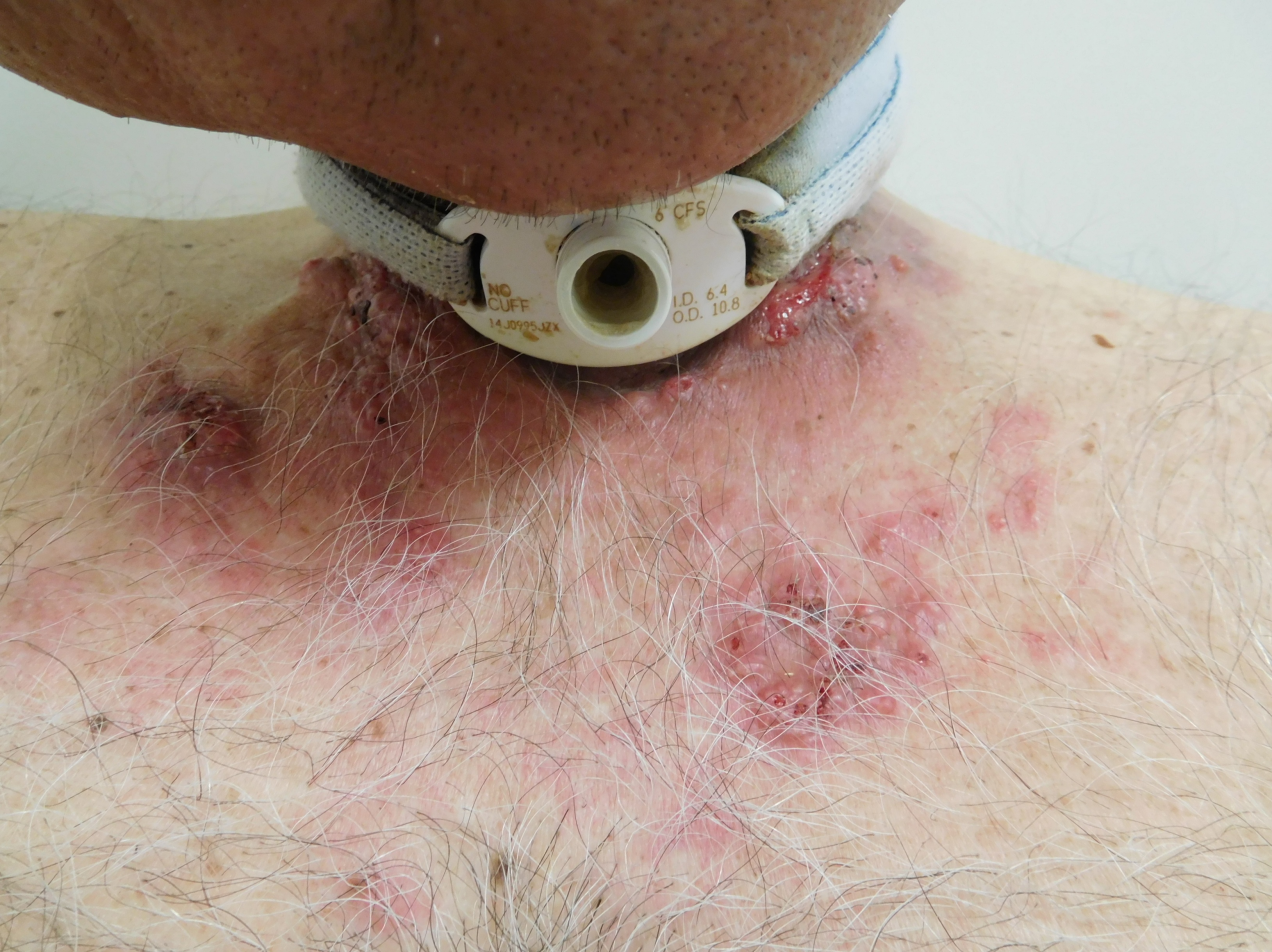
Central view of cutaneous metastases from tonsillar squamous cell carcinoma. Multiple erythematous plaques composed of individual and confluent papules are seen on the neck and proximal chest. Ulcerated and crusted nodules are seen on the neck.

**Figure 2:**
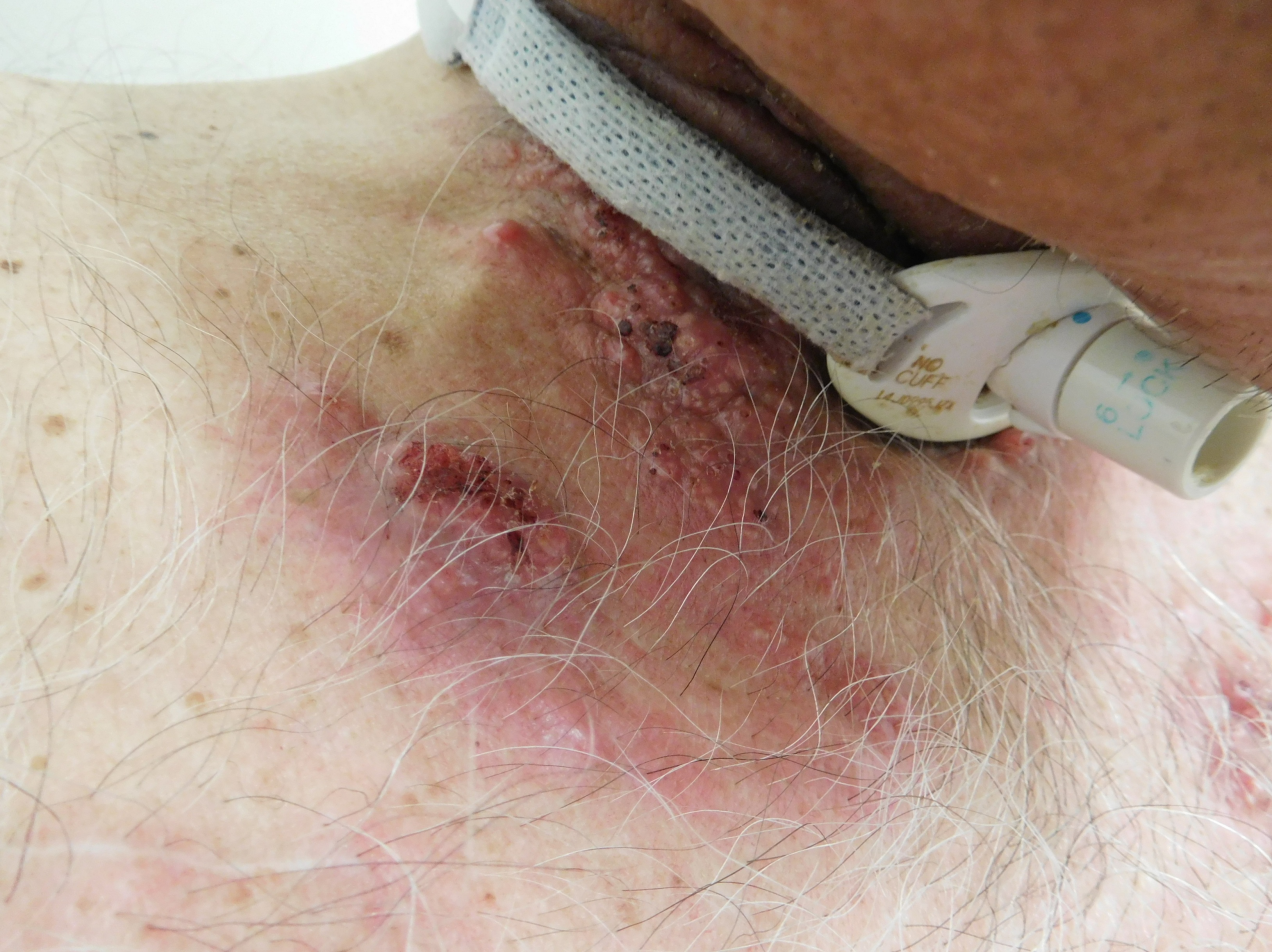
Right-sided view of cutaneous metastases from tonsillar squamous cell carcinoma. Multiple confluent papules and erythematous plaques are seen beneath the patient’s tracheostomy collar and also on the proximal right chest in the area of his prior radiation therapy port.

**Figure 3:**
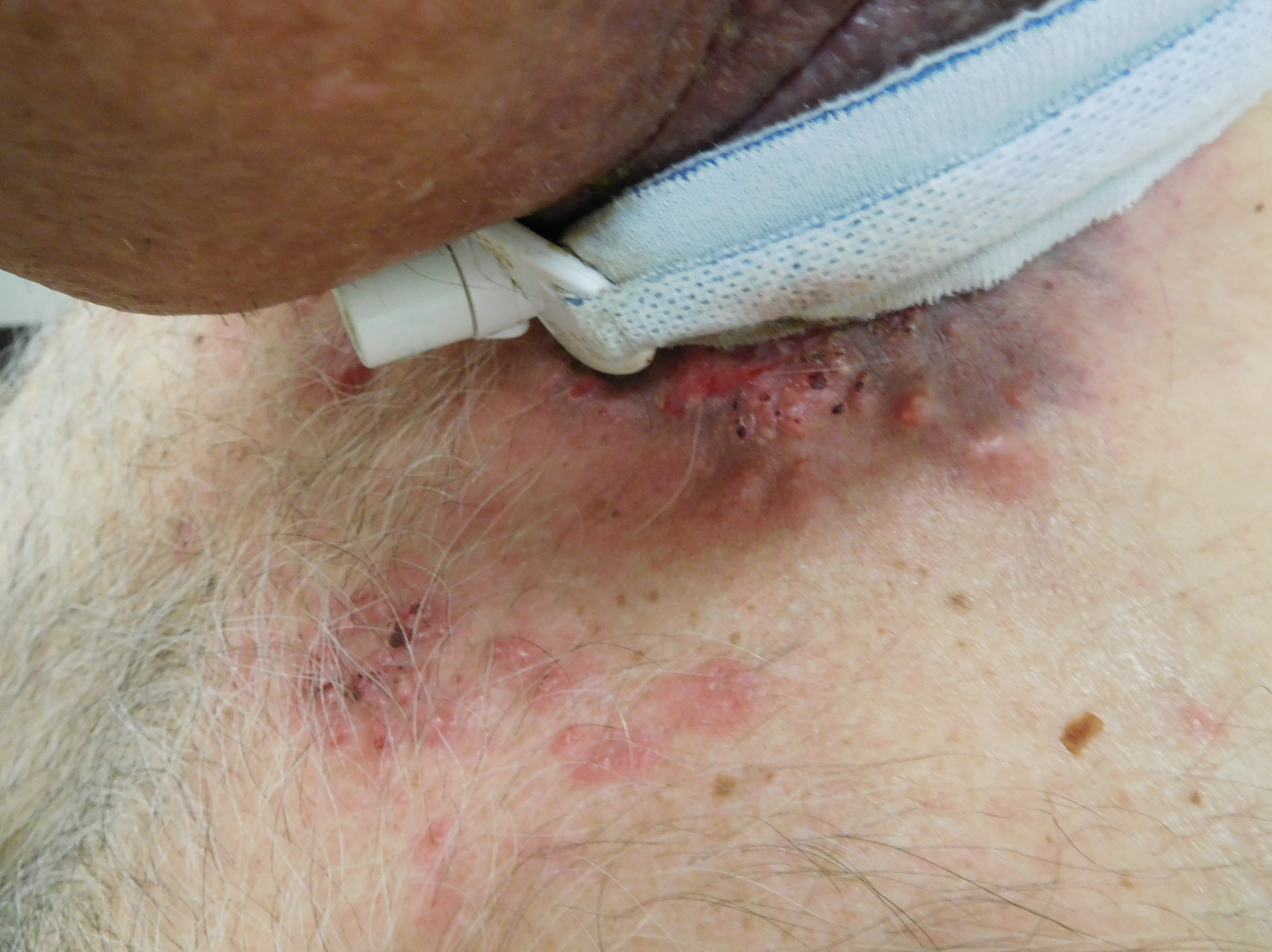
Left-sided view of cutaneous metastases from tonsillar squamous cell carcinoma. Erythematous plaques and ulcerated nodules are observed on the left side of the patient’s neck adjacent to his tracheostomy collar, along with papules on the left proximal chest in the area of his prior radiation port.

**Figure 4:**
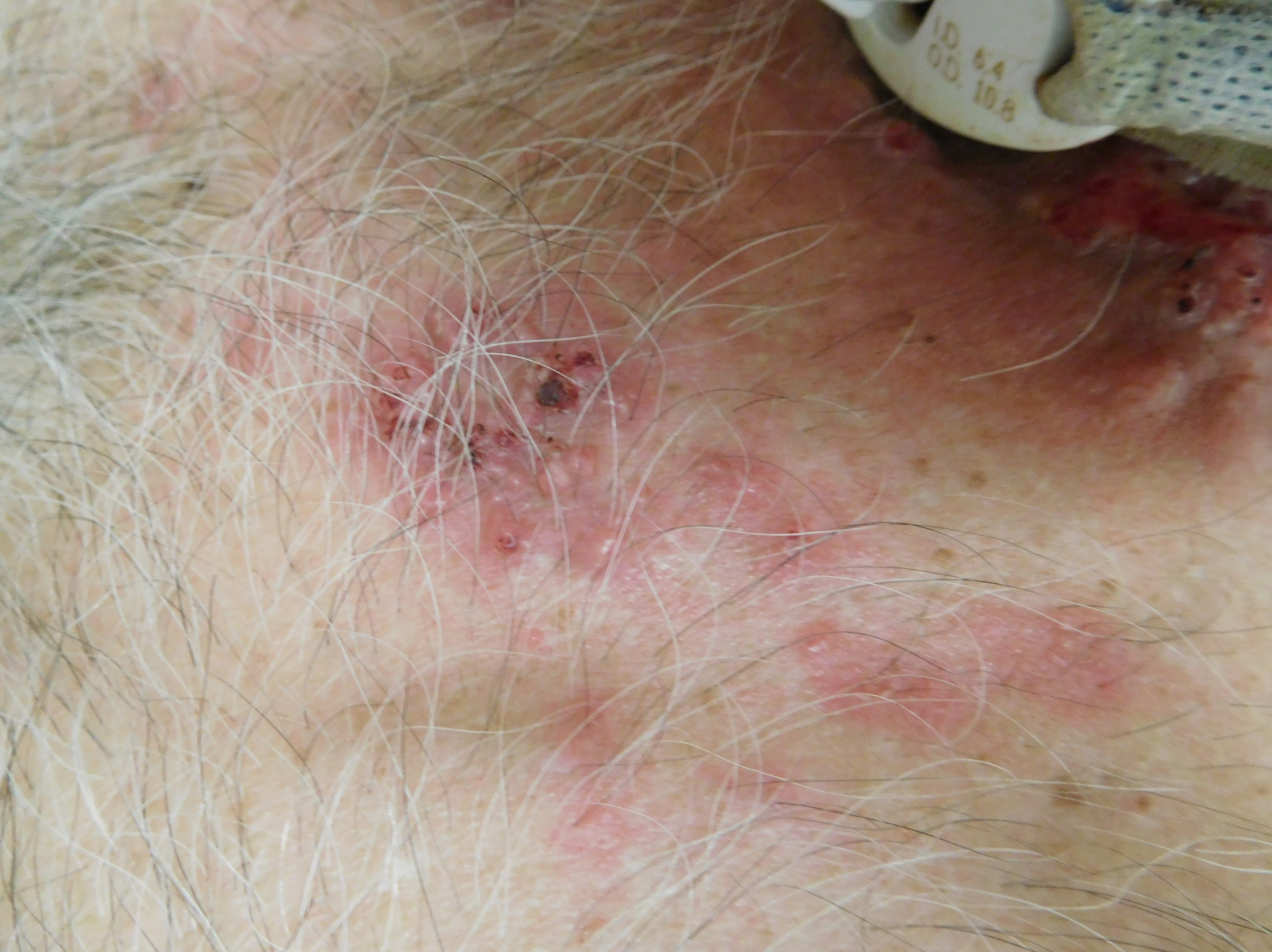
Closer view of tonsillar squamous cell carcinoma cutaneous metastases on the proximal chest. These erythematous papules on the chest were biopsied.

Cutaneous examination of the patient’s neck and proximal chest revealed multiple erythematous plaques, up to 6x4 cm, consisting of individual and confluent papules; ulcerated and crusted nodules were present on the patient’s neck within the patient’s prior radiation port and also beneath his tracheostomy collar (Figures 1-4). No vesicles or bullae were seen. Odor was noted, but no purulent discharge was observed. The clinical differential diagnosis included cellulitis, cutaneous metastases from tonsil carcinoma, and varicella zoster virus infection. Initial evaluation included bacterial culture and two punch biopsies from the left chest.

The bacterial culture revealed Staphylococcus aureus with sensitivity to cephalexin. Both biopsies showed nodular aggregates of atypical keratinocytes in the superficial dermis with central necrosis and multiple dyskeratotic cells; they did not extend from the overlying epidermis. Immunohistochemistry with p40 and cytokeratin 5/6, both of which are markers for squamous cell carcinoma, were positive. Correlation of the clinical lesions and the pathology findings established the diagnosis of metastatic squamous cell carcinoma.

The patient was started on systemic cephalexin 500 mg four times daily and topical 2% mupirocin ointment. The patient noted that his neck lesions were less painful and that the odor had disappeared. He continued these medications for 30 days.

The pathology results were discussed with the patient. He maintained his decision not to seek any further cancer treatment. He is followed by the outpatient palliative care service and intends to transition to hospice care when appropriate.

## Discussion

Tonsillar cancer is uncommon. The most common type is squamous cell carcinoma (75%), while lymphoma accounts for the majority of the remaining cases [6]. Cutaneous metastases from tonsillar squamous cell carcinoma are rare; to the best of our knowledge, including our patient, only four patients have been described in the literature (Table 1) [3-5]. Notably, all four individuals were men.

**Table 1:** Summary of characteristics from patients with cutaneous metastases from squamous cell carcinoma of the tonsil Abbreviations in order of appearance: Mets, metastases; PO, periocular; SCC, squamous cell carcinoma; XRT, radiation therapy; %, percent; MTX, methotrexate; FNA, fine needle aspiration.

Case	Age at initial diagnosis (years); race; sex	Time to skin mets (months)	Location of skin mets	Morphology of skin mets	Pathology of skin mets	Treatment after skin mets diagnosis	Follow-up	Reference
1	40; not reported; man	25	Left temple, PO, and malar face	Erythematous, individual and confluent, papules and nodules	Metastatic SCC consistent with primary disease	Palliative XRT	50% size reduction after four months	4
2	55; not reported; man	6	Scalp, left thigh	Ulcerated nodules	Moderately differentiated SCC consistent with primary disease	Palliative MTX	No extensive follow-up reported, but patient tolerated chemotherapy	3
3	55; White; man	54	Neck (previous XRT port), proximal chest	Erythematous, individual and confluent, papules, plaques, and nodules; ulcerated nodules	Atypical nodular aggregates of keratinocytes, central necrosis, and multiple dyskeratotic cells consistent with metastatic SCC	Cancer treatment refused	Followed by palliative care service with plans to transition to hospice when appropriate	Current report
4	70; n​ot reported; man	16	Right forehead	Plaques and nodules	FNA of fluid from lesion revealed squamoid cells with pleomorphic nuclei along with individual cell keratinization consistent with metastatic SCC	Palliative XRT offered but refused	Patient died two days after skin mets diagnosis	5

Human papilloma virus (HPV) has been associated with squamous cell carcinoma; specifically, HPV-16 and HPV-18 have oncogenic genotypes. Although HPV has been implicated in the pathogenesis of tonsillar malignancy, few studies have shown a causal relationship [6]. Viral studies of the tumors of the four men with cutaneous metastases from their tonsillar squamous cell carcinoma had not been performed.

Cutaneous metastasis from tonsillar squamous cell carcinoma was initially reported in 2002 [4]. A 40-year-old man with a history of smoking presented with dysphagia of three months' duration along with bilateral neck swelling for one month. Biopsy confirmed well-differentiated squamous cell carcinoma of the tonsils, and the patient was treated with radiation therapy for seven weeks. He remained disease-free for 25 months. He then presented with individual and confluent papules and nodules on the left periocular, temple, and malar cheek with erythematous papules and plaques. Fine needle aspiration revealed metastatic squamous cell carcinoma. The patient was treated with palliative radiation therapy, and the swelling was reduced in size by 50% at the four-month follow-up [4].

The second patient described with cutaneous metastasis from tonsillar squamous cell carcinoma was reported in 2015 [5]. A 70-year-old man with a history of smoking presented with two months of dysphagia and weight loss. Biopsy of the left tonsil revealed moderately differentiated squamous cell carcinoma. He was treated with radiation therapy and was disease-free for 16 months. He then noted “swelling of the right frontoparietal region without any overlying skin changes”. The published images of his clinical lesions demonstrated nodules and plaques. He was in poor general condition: wheelchair-bound and malnourished. Fine needle aspiration revealed cutaneous metastasis of squamous cell carcinoma. The patient refused palliative radiation therapy and died two days after cutaneous metastasis was diagnosed [5].

The third patient with cutaneous metastasis from tonsillar squamous cell carcinoma was reported in 2016 [3]. A 55-year-old man with a history of smoking presented with dysphagia, throat pain, and weight loss for three months. Biopsy of the right tonsil revealed moderately differentiated squamous cell carcinoma, and the patient was treated with cisplatin chemotherapy with concurrent radiation therapy for six weeks. After six months, the patient presented with four ulcerated nodules: three on his scalp and one on his left thigh. Biopsy of the skin lesions demonstrated squamous cell carcinoma. The patient received palliative treatment with methotrexate [3].

Cutaneous metastases from tonsillar squamous cell carcinoma have been described in four men (Table 1) [3-5]. Three of the patients had a history of smoking. Although there is an established association between alcohol use and smoking with tonsillar malignancy, some individuals with carcinoma of the tonsil – such as our patient – lack one or both of these risk factors [6].

Tonsillar squamous cell carcinoma, in the men who subsequently developed cutaneous metastases, was diagnosed when they were between 40 to 70 years of age, with a median age of 55 years. Skin lesions never preceded the diagnosis of tonsillar carcinoma; cutaneous metastases manifested six to 54 months after the diagnosis of malignancy, with a median interval of 21 months.

All of the patients with cutaneous metastasis presented with skin lesions on their head or neck (Table 1) [3-5]; in addition, distant metastasis to the thigh was also noted in one man [3]. Cutaneous metastases result from three possible mechanisms. The local spread of cancer to overlying skin may result from direct extension through contiguous tissue planes. More distant spread of malignancy is possible through either dermal lymphatics or blood vessels [7].

In our patient, cutaneous metastases presented on the neck and proximal chest, which was the former site of his radiation therapy port. A recent review of cutaneous metastases within radiation ports noted that breast cancer was the most common tumor to metastasize to sites of prior radiation therapy [8]. Moreover, radiation port metastases have also been described in patients with other primary malignancies [8]. It has been hypothesized that the appearance of the cutaneous metastases within radiation ports may result from the development of cutaneous immunocompromised districts [9]; ionizing radiation damages the skin and alters the immune response, thereby rendering the patient vulnerable to an isoradiotopic response that manifests as cutaneous metastasis [9-10].

The morphology of the cutaneous metastases from tonsillar squamous cell carcinoma is variable. In a review of over 4,000 patients with metastatic carcinoma, cutaneous metastases was observed to manifest as bullous, cicatricial, and inflammatory lesions, though nodules were the most common presentation [2]. Cutaneous metastases from tonsillar squamous cell carcinoma presented as erythematous, intact and ulcerated, individual and confluent, papules, plaques, and nodules. The cutaneous metastases may raise suspicion for other conditions; our patient’s skin lesions were suspicious for a varicella zoster virus infection.

Treatment of cutaneous metastasis from tonsillar squamous cell carcinoma was palliative [3-5]. Two patients were offered radiation therapy: one man refused treatment, while the other man experienced a 50% reduction in the size of his skin metastases after four months [4-5]. Another patient was treated with methotrexate; his response to therapy was not reported [3]. Our patient had refused all cancer treatment three months prior to the diagnosis of his cutaneous metastasis; he maintained this decision after learning that he had developed biopsy-confirmed cutaneous metastases from his tonsillar squamous cell carcinoma.

Generally, cutaneous metastasis is thought to be a poor prognostic marker [4]; mean survival from the diagnosis of cutaneous metastases in head and neck cancers is approximately seven months [3]. Indeed, the prognosis of patients with cutaneous metastases from tonsillar squamous cell carcinoma is very unfavorable. The detection of metastatic skin tumors indicates progression of the malignancy with the option of palliative treatment; mortality and morbidity may hinge on a patient’s functional status at the time of diagnosis of cutaneous metastasis, as well as the willingness of the patient to pursue palliative therapy. Specifically, in our review of four patients, one man died within two days of his diagnosis of cutaneous metastasis; however, he was very ill with poor general health when diagnosed with skin metastasis [5]. The other patient whose follow-up was described experienced improvement after four months of palliative radiation therapy [4]. Our patient is being managed by the outpatient palliative care service to maximize quality of life; he anticipates a transition to hospice care when appropriate. 

## Conclusions

Cutaneous metastasis from squamous cell carcinoma of the tonsil is rare. To the best of our knowledge, in addition to our patient, only four cases have been reported. Intriguingly, all four patients were men. The metastases presented within six months to four and a half years after diagnosis of their tonsillar malignancy. Cutaneous metastases of tonsillar squamous cell carcinoma may present on the skin overlying the visceral malignancy, at nearby sites, such as the chest and the head, or at distant locations, such as the extremities. The metastases are postulated to spread either by direct extension or hematogenously and/or within lymphatics. The morphology of cutaneous metastases is variable; therefore, new lesions in a patient with an established diagnosis of tonsillar squamous cell carcinoma should be considered for biopsy. Thus far, the treatment in all of the men has been palliative chemotherapy or radiation therapy. Data on prognosis is limited since extensive follow-up for these patients is limited. However, the invasive nature of the disease portends poor outcomes, and cutaneous metastases are considered a poor prognostic marker. Treatment should be individualized to the patient’s condition and preferences.
